# Giant Congenital Melanocytic Nevi in a Pakistani Newborn

**DOI:** 10.7759/cureus.15210

**Published:** 2021-05-24

**Authors:** Hina Mumtaz Hashmi, Nazia Shamim, Vinod Kumar, Sidra Idrees

**Affiliations:** 1 Pediatrics and Child Health, Aga Khan University Hospital, Karachi, PAK

**Keywords:** giant congenital melanocytic nevi, melanoma, satellite lesions, neurocutaneous melanosis, nevomelanocytes

## Abstract

Congenital melanocytic nevi arise from overgrowth or disrupted migration of melanocyte precursor in the neural crest. They are also known as coat-sleeve, stocking, bathing trunk or garment nevi. The colour ranges from brown to black, with the lesions presenting as flat to raised nevi. Lesions presenting at birth with a diameter greater than 20cm are labelled giant congenital melanocytic nevi. Risk increases with an increase in the number of satellite lesions near the giant nevus. Management includes regular clinical follow-up monitoring of changes in the lesion and surgical procedures in cases with risk of melanoma and psychological support. The purpose of this case presentation is to describe a rare issue of giant congenital melanocytic nevi in a newborn, along with a literature review and discussion on possible management options.

## Introduction

Congenital melanocytic nevi (CMN) are skin lesions characterized histologically by benign proliferations of nevomelanocytes [1]. They are commonly found on the back and thigh with a brown to black colour presenting at birth or within the first few weeks of life, with few studies demonstrating presentation as late as two years of age [2,3]. CMN are usually categorized depending on the adult size of maximum diameter of the lesion into small, medium, large or giant nevi. Small CMNs are usually <1.5cm, medium 1.5-19.9cm, large 20-40cm and giant >40cm in maximal diameter [4,5]. This classification is also accepted and stated by the University of New York [6]. The incidence of giant congenital melanocytic nevi (GCMN) is < 1 in 20,000 neonates [3,6]. Even with the rare presentation, the follow up of this lesion is important as it is linked with severe complications affecting the skin (malignant melanoma), nervous system (Neurocutaneous melanosis) and psychosocial implications, including distress for both the parents and the child due to not only the appearance of the lesion but also because of the difficult and challenging management and outcome [3].

A useful factor for prognosis is the predicted size of the lesions in adulthood. GCMN or the lesions >40cm are the greatest at risk for malignancy, approximately 6%, and large lesions (20-40cm) carry a 4-6% [2,7]. Fifty percent of the melanomas in GCMN develop by the age of two and 80% by seven [2]. Small nevi are rarely seen to progress to melanoma [7]. The conventional treatment includes surgical removal of the primary lesion, but other options are being explored. We present a rare case of GCMN along with a review of literature and discussion. As per our knowledge, this is the first case of GCMN from Pakistan.

## Case presentation

**Figure 1:**
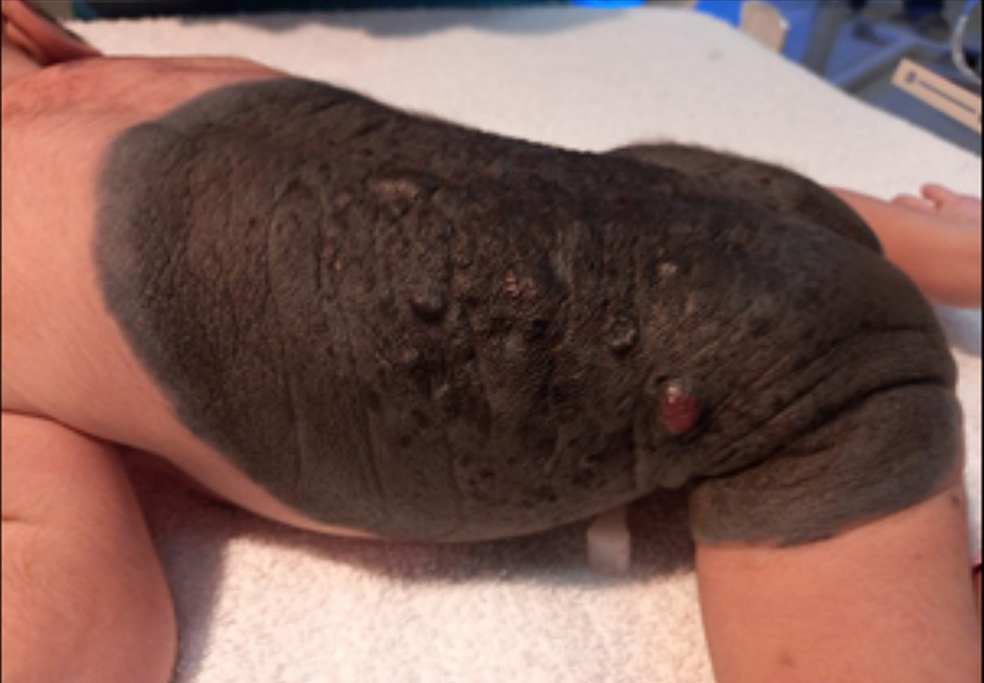
Giant congenital melanocytic nevus over back with nodules and pigmentation

**Figure 2:**
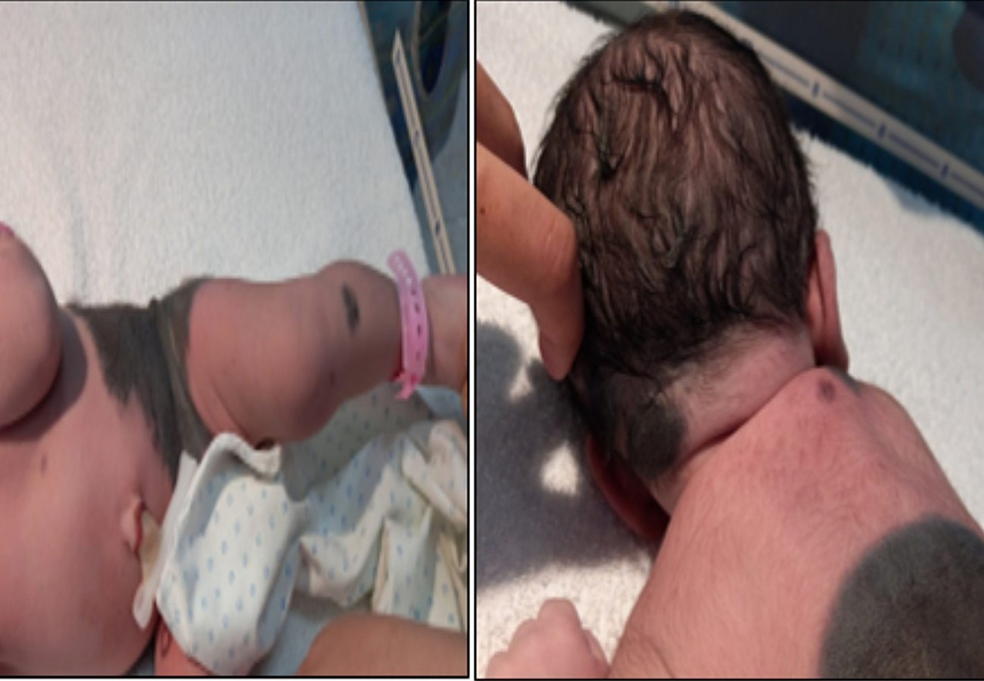
Multiple satellite lesions and small lesions over nape of neck and upper back

We present a case of a male neonate born to healthy parents. This was the second child of a normally progressing pregnancy delivered in a secondary hospital via cesarean section due to a transverse lie with good Appearance, Pulse, Grimace, Activity, and Respiration (APGAR). There were no abnormalities relating to pregnancy or delivery. Antenatal scans, including anomaly scan, were unremarkable. There was no family history of any dermatological disease. The parents were non-consanguineous. On examination, a giant melanocytic nevus was present on the back, measuring 20cm on the vertical axis and 21cm on the horizontal axis with irregular borders with dark brown colour and pigmentation over the whole lesion (Figure 1). A nodular lesion was evident in the left portion of the giant nevus, along with some hair on the lesion. The large nevus was accompanied by multiple satellite lesions approximately 26 in number on the body, including one on the forehead along with three small lesions over the left shin, right upper back and left nape of the neck, with the largest measuring 1.5cm x 3cm on the nape of the neck (Figure 2). There was no other associated congenital abnormality that was evident. Baseline ultrasound head and sacrum were done but were unremarkable. Dermatological opinion was taken during the stay in the hospital and was planned for follow up. A neurological and genetics follow up was also given. Ophthalmological examination at birth revealed positive red reflex bilaterally. During the hospital stay, the baby remained well and counselled the family regarding disease presentation and further investigation on follow up.

## Discussion

The lesions in CMN are pigmented cutaneous lesions formed by a combination of epidermally and dermally derived nevus cells. These may also be referred to as giant hairy nevi due to hair growth in excess. An equal gender distribution has been reported in the literature. [2]

Some studies report that GCMN may result from spontaneous mutations during fetal development, but genetic inheritance with familial presentation has been observed. Chromosomal rearrangements involving 1p, 12p and 19p have been shown in a culture of melanocytes from the nevus [2]. Literature reports 14 somatic mutations in one study noted in 21 patients with CMN, with 57% of the lesions showing mutations in the NRAS gene [8]. Another study found a gain of function mutation in the NRAS gene associated with GCMN, ultimately leading to an abnormal proliferation of melanoblasts [9]. Some literature reports BRAF-activating mutations (single-nucleotide variants and fusions) activate the MAPK pathway [5].

A protein, HGF/SF (hepatocyte growth factor/ scatter factor), is reviewed by some researchers to be the reason for neuroectodermal cell scattering and migration lead to presence all over the body. Due to the excess or abnormal protein in some cells, extra pigment and abnormal skin cells develop called the nevus cells [2].

Malignant melanoma, a complication of GCMN, progresses in the first five years of life, whereas those arising from small and medium-sized nevi occur after puberty. Histopathology is considered the gold standard for the diagnosis of malignant melanoma. Diagnosis of melanoma in cases of GCMN are made with excisional biopsy with punch biopsies considered for exposed sites like face, palms and soles [10]. Of the complications seen in previous studies, the most serious and rare is neurocutaneous melanosis (NCM). It is characterized by the presence of melanocytic proliferations in the central nervous system (CNS), which may be benign or malignant [3,11].

Along with NCM, Arnold Chiari type I, Dandy-Walker malformation, and other structural malformations including spinal dysraphism, cerebellar astrocytoma and arachnoid cysts have been seen in association with CMN [3]. Investigations done to evaluate the extension of disease and CNS involvement ideally at the age of 4-6 months includes radiographic imaging by MRI to check for any melanocytic deposition in the CNS. [2]

Initial management includes regular follow up with at least an annual examination of the lesions for the initial three years with 2-5 yearly follow up later on. Literature reports surgery as the mainstay of treatment for CMN [12]. Although impractical at initial presentation, especially for small lesions, the decision for surgical excision is considered at six months of age, preferably after assessing neurological extension and CNS involvement, along with the issues of technical difficulties and uncertainty regarding effective prophylaxis against the development of melanoma [2,3].

Procedures used in surgical treatment include reconstruction with skin grafting, local rotation flaps and serial excision bearing in mind that leptomeningeal involvement will not eliminate the risk of melanoma even after surgical excision [2]. To aid in psychological support and cosmetic purposes, partial removal of GCMN by skin curettage, dermabrasion, laser and chemical peels have been used. The procedures used in the surgical treatment include serial excision and reconstruction with skin grafting and tissue expansion local rotation flaps and free tissue transfer [3].

Recent advances include the use of carbon dioxide and ruby laser, Er: YAG and Q-switched, respectively, for selective treatment of deep pigmentations and resurfacing [2]. With studies showing a link to mutations in the NRAS gene, the role of inhibitors of NRAS and high hydrostatic pressure inactivating the nevus tissue is also being considered [9].

Recurrence has been observed in cases even after the removal of the lesion. In some instances, melanomas even arise from different sites other than the original nevi requiring a regular examination to identify any change in a lesion or possible development of malignancy or complications [7].

## Conclusions

Evaluation of GCMN is very challenging as the nevi change with time. Any change noted in size, shape or colour on follow up should warrant further investigation. A multidisciplinary team approach, including a general paediatrician, dermatologist, neurologist, plastic surgeon, geneticist, and psychologist, is ideal for managing these cases as even removing lesions prophylactically does not eliminate the risk of recurrence of melanoma. Such patients require lifelong regular follow up and detailed examination to detect any malignancy.

## References

[1] Krengel S, Scope A, Dusza SW, Vonthein R, Marghoob AA (2013). New recommendations for the categorization of cutaneous features of congenital melanocytic nevi. J Am Acad Dermatol.

[2] Das SK, Amarendra M, Subudhi M (2012). Giant congenital melanocytic nevi: a case report. J Clin Diagn Res.

[3] Viana ACL, Gontijo B, Bittencourt FV (2013). Giant congenital melanocytic nevus. An Bras Dermatol.

[4] Ibrahimi OA, Alikhan A, Eisen DB (2012). Congenital melanocytic nevi: where are we now? Part II. treatment options and approach to treatment. J Am Acad Dermatol.

[5] Mir A, Agim NG, Kane AA, Josephs SC, Park JY, Ludwig K (2019). Giant congenital melanocytic nevus treated with trametinib. Pediatrics.

[6] Huang WL (2019). Is it possible to treat giant congenital hairy melanocytic nevus clinically?. J Clin Case Rep Trials.

[7] McLaughlin MR, O’Connor NR, Ham P. (2008). Newborn skin: part II. birthmarks. Am Fam Physician.

[8] da Silva VM, Martinez-Barrios E, Tell-Marti G (2019). Genetic abnormalities in large to giant congenital nevi: beyond NRAS mutation. J Invest Dermatol.

[9] Meshram GG, Kaur N, Hura KS. (2018). Giant congenital melanocytic nevi: an update and emerging therapies. Case Rep Dermatol.

[10] Kumari M, Singh M, Punhani P (2020). Malignant melanoma in a child with giant congenital melanocytic nevus and satellite flekers: a rare entity. Diagn Cytopathol.

[11] Moustafa D, Blundell AR, Hawryluk EB (2020). Congenital melanocytic nevi. Curr Opin Pediatr.

[12] Fahradyan A, Wolfswinkel EM, Tsuha M (2019). Cosmetically challenging congenital melanocytic nevi. Ann Plast Surg.

